# Epstein-Barr Virus-Specific CD8 T Cells Selectively Infiltrate the Brain in Multiple Sclerosis and Interact Locally with Virus-Infected Cells: Clue for a Virus-Driven Immunopathological Mechanism

**DOI:** 10.1128/JVI.00980-19

**Published:** 2019-11-26

**Authors:** Barbara Serafini, Barbara Rosicarelli, Caterina Veroni, Gina Adriana Mazzola, Francesca Aloisi

**Affiliations:** a Department of Neuroscience, Istituto Superiore di Sanità, Rome, Italy; b S.C. Immunogenetica e Biologia dei Trapianti U, Azienda Ospedaliero Universitaria Città della Salute e della Scienza di Torino, Turin, Italy; Northwestern University

**Keywords:** brain, cytotoxicity, EBV-specific CD8 T cells, Epstein-Barr virus, immunopathology, multiple sclerosis

## Abstract

Epstein-Barr virus (EBV) is a ubiquitous herpesvirus strongly associated with multiple sclerosis (MS), a chronic inflammatory disease of the central nervous system (CNS). However, the mechanisms linking EBV infection to MS pathology are uncertain. Neuropathological and immunological studies suggest that a persistent EBV infection in the CNS can stimulate a CD8 T-cell response aimed at clearing the virus but inadvertently causing CNS injury. Inasmuch as *in situ* demonstration of EBV-specific CD8 T cells and their effector function is missing, we searched for EBV-specific CD8 T cells in MS brain tissue using the pentamer technique. Postmortem brain samples from 12 donors with progressive MS and known HLA class I genotype were analyzed. Brain sections were stained with HLA-matched pentamers coupled with immunogenic peptides from EBV-encoded proteins, control virus (cytomegalovirus and influenza A virus) proteins, and myelin basic protein. CD8 T cells recognizing proteins expressed in the latent and lytic phases of the EBV life cycle were visualized in white matter lesions and/or meninges of 11/12 MS donors. The fraction (median value) of CD8 T cells recognizing individual EBV epitopes ranged from 0.5 to 2.5% of CNS-infiltrating CD8 T cells. Cytomegalovirus-specific CD8 T cells were detected at a lower frequency (≤0.3%) in brain sections from 4/12 MS donors. CNS-infiltrating EBV-specific CD8 T cells were CD107a positive, suggesting a cytotoxic phenotype, and stuck to EBV-infected cells. Together with local EBV dysregulation, selective enrichment of EBV-specific CD8 T cells in the MS brain supports the notion that skewed immune responses toward EBV contribute to inflammation causing CNS injury.

**IMPORTANCE** EBV establishes a lifelong and asymptomatic infection in most individuals and more rarely causes infectious mononucleosis and malignancies, like lymphomas. The virus is also strongly associated with MS, a chronic neuroinflammatory disease with unknown etiology. Infectious mononucleosis increases the risk of developing MS, and immune reactivity toward EBV is higher in persons with MS, indicating inadequate control of the virus. Previous studies have suggested that persistent EBV infection in the CNS stimulates an immunopathological response, causing bystander neural cell damage. To verify this, we need to identify the immune culprits responsible for the detrimental antiviral response in the CNS. In this study, we analyzed postmortem brains donated by persons with MS and show that CD8 cytotoxic T cells recognizing EBV enter the brain and interact locally with the virus-infected cells. This antiviral CD8 T cell-mediated immune response likely contributes to MS pathology.

## Abstract (precis)

EBV establishes a lifelong and asymptomatic infection in most individuals and more rarely causes infectious mononucleosis and malignancies, like lymphomas. The virus is also strongly associated with MS, a chronic neuroinflammatory disease with unknown etiology. Infectious mononucleosis increases the risk of developing MS, and immune reactivity toward EBV is higher in persons with MS, indicating inadequate control of the virus. Previous studies have suggested that persistent EBV infection in the CNS stimulates an immunopathological response, causing bystander neural cell damage. To verify this, we need to identify the immune culprits responsible for the detrimental antiviral response in the CNS. In this study, we analyzed postmortem brains donated by persons with MS and show that CD8 cytotoxic T cells recognizing EBV enter the brain and interact locally with the virus-infected cells. This antiviral CD8 T cell-mediated immune response likely contributes to MS pathology.

## INTRODUCTION

Multiple sclerosis (MS) is the most common chronic inflammatory disease of the central nervous system (CNS) and the leading cause of nontraumatic neurological disability in young adults. Leukocyte infiltration, demyelination, neurodegeneration, and reactive gliosis in the CNS are the histopathological hallmarks of MS, leading to progressive deterioration of motor, sensory, and cognitive functions (1). The precise etiology of MS and whether it is self or nonself antigens that trigger persistent CNS inflammation is not yet clear. The current belief is that MS develops in a genetically susceptible host upon interaction with environmental factors (2). More than 200 susceptibility genes have been identified, with the HLA DRB1*1501 allele conferring the strongest risk for MS (3). The most common environmental risk factors are sunlight exposure/vitamin D level, cigarette smoking, and infectious agents (2). Among the latter, Epstein-Barr virus (EBV), a human DNA herpesvirus infecting about 95% of the adult population worldwide, is the main candidate culprit; no other pathogen shows such a strong association with MS (4, 5).

EBV infection commonly occurs early in life, is asymptomatic, and is carried as a latent, lifelong infection of B cells that is tightly controlled by the host’s immune system (6). However, in some circumstances the virus-host balance is disturbed and the pathogenic potential of EBV can manifest. EBV is etiologically linked to a variety of human tumors, including lymphomas and nasopharyngeal carcinoma, and to diseases like infectious mononucleosis, the genetically acquired X-linked lymphoproliferative disease, and chronic active EBV infection, which are caused by exaggerated immune responses to the virus resulting in bystander tissue damage (7).

The association of EBV with MS is mainly supported by large-scale studies that have consistently shown virtually 100% EBV seroprevalence in adult MS, higher anti-EBV antibody titers, particularly for EBNA1 IgG, in MS patients than in healthy subjects (5, 8–10), and an increased risk of developing MS after infectious mononucleosis (11). It has been shown that EBV infection is required for MS development (12) and that the rise in anti-EBV antibody titers occurs already several years (up to 20) before MS diagnosis (13–15), suggesting a causative role of EBV in MS. Elevated CD4 and CD8 T cell responses to EBV in MS patients, particularly during disease flares, are consistent with abnormal EBV infection in MS (16–20). Studies have also shown impaired EBV-specific CD8 T cell responses in MS patients (21–23) and a decreased frequency or functionality of EBV-specific CD8 T cells with increasing disease duration (18, 22, 23), suggesting T-cell exhaustion due to a persistently active infection. Since EBV establishes latency in memory B cells (6), confirmation of the beneficial effects of B-cell-depleting anti-CD20 monoclonal antibodies (MAbs) in large MS cohorts (24, 25) has reinforced the association between EBV and MS and the need for studies to elucidate the mechanisms linking EBV infection to MS pathology (26, 27).

Molecular mimicry between viral and CNS myelin antigens (28, 29) and EBV-mediated induction of a putative autoantigen, the small stress protein alpha B-crystallin (30), have been proposed as possible mechanisms underlying the EBV-MS link. However, the available evidence is still inconclusive. Immortalization of autoreactive B cells by EBV remains hypothetical (31), especially when one takes into account the unproven role of autoantibodies in MS and the fact that the rapid clinical effect of B-cell-depleting drugs in MS is not accompanied by a reduction in antibody production and intrathecal Ig synthesis (32). This rules out the involvement of humoral immunity in the therapeutic effect. Evidence also is missing that EBV is preferentially harbored in autoreactive B cells (33). Another possibility is that impaired immune responses to EBV at primary infection results in increased viral load (34), allowing migration of activated, circulating EBV-infected B cells into the CNS. Due to its ability to stimulate B-cell proliferation (6), EBV would promote intrathecal B-cell expansion and maturation, a hallmark of MS (1). A CNS-secluded, low-grade, and chronically active EBV infection would stimulate an immune response that promotes inflammation and inadvertently causes damage to the CNS (7). This pathogenic model is supported by our studies in postmortem MS brain tissue showing a dysregulated, predominantly latent EBV infection in CNS-infiltrating B cells, particularly those forming ectopic B-follicle-like structures in the meninges (35–38), and viral reactivation in CNS-infiltrating plasmablasts/plasma cells (19, 35, 38). EBV latent and lytic transcripts were also detected in laser-cut CNS immune infiltrates (19, 36, 39). Most importantly, we have shown that EBV dysregulation in the CNS is characteristic of MS and is not observed in patients with other infectious and noninfectious inflammatory diseases of the CNS (35, 36). Abnormal EBV infection in the MS brain has been confirmed in other independent studies (40–42), while several researchers failed to detect EBV in MS brain lesions (43–46). Such discrepancies across studies may be explained by differences in sample selection and methods/tools to detect EBV (47, 48).

Although an EBV-induced immunopathological model of MS is biologically plausible, it needs to be substantiated by a more detailed understanding of the immune components involved. Cytotoxic CD8 T cells play an important role in controlling EBV infection (49), and it is well established that CD8 T cells represent the predominant T-cell population in MS brain inflammatory lesions (50, 51). It was also shown that MS brain-infiltrating CD8 T cells undergo clonal expansion (52) and express a cytotoxic effector phenotype (19, 35, 38, 53) indicating *in situ* activation. Several studies have demonstrated selective enrichment of EBV-specific CD8 T cells but not CD8 T cells recognizing cytomegalovirus (CMV) or candidate MS-associated autoantigens, in the cerebrospinal fluid (CSF) of MS patients (54–57), suggesting activation of a localized cytotoxic T-cell response toward EBV.

Despite intimate contacts between cytotoxic CD8 T cells and EBV-infected cells being visualized in the MS brain (19, 35, 38, 58), direct demonstration of the presence and effector function *in situ* of EBV-specific CD8 T cells is missing. This issue can be tackled by using fluorochrome-labeled, major histocompatibility complex (MHC) class I peptide multimers (tetramers or pentamers), which allow the distinguishing of antigen-specific from total CD8 T cells in appropriately processed human tissues (59–61). In this study, we used postmortem brain tissue donated by persons with MS and *in situ* pentamer staining to (i) characterize the EBV antigens recognized by CNS-infiltrating CD8 T cells, (ii) compare the frequency of EBV-specific CD8 T cells with that of CD8 T cells recognizing other common viruses or a putative myelin autoantigen, and (iii) study the cytotoxic effector function of CNS-infiltrating, EBV-specific CD8 T cells and their spatial proximity to virus-infected B cells/plasma cells.

## RESULTS

### Neuropathological characteristics of MS brain samples and visualization of EBV-specific CD8 T cells in brain sections.

**FIG 1:**
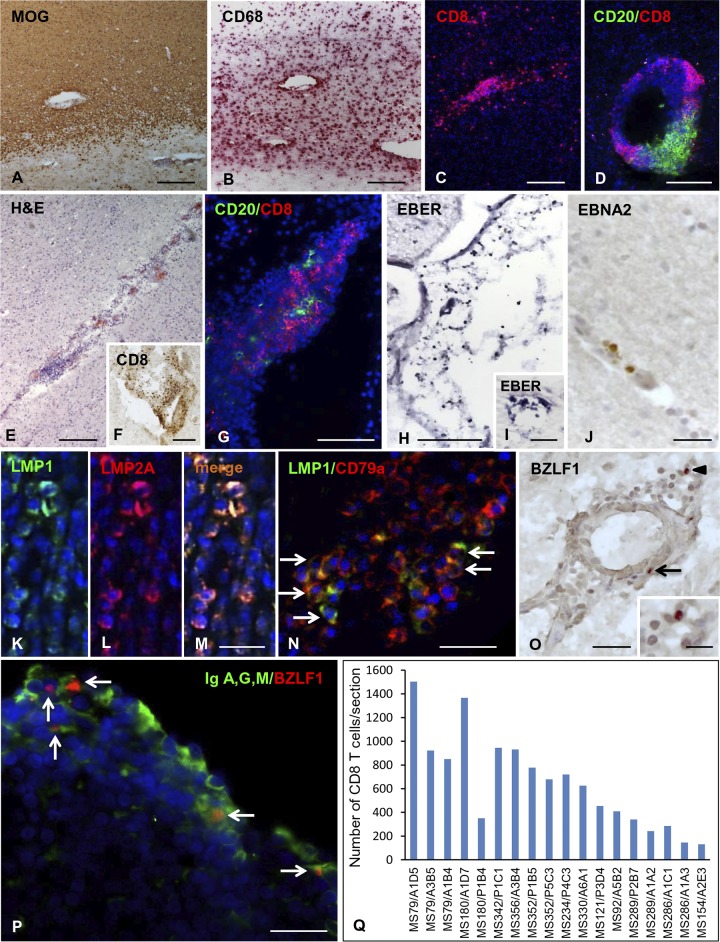
Neuropathological characteristics, EBV infection, and CD8 T cell infiltration in the MS brain. Stainings of brain sections from 3 MS donors included in this study are shown. (A to D, donor MS121) Actively demyelinating WM lesion characterized by myelin loss (A, MOG immunostaining), foamy macrophages (B, CD68 immunostaining), and prominent CD8 T cell infiltration (C, CD8 immunostaining). A large perivascular cuff with high-density B cells and CD8 T cells is shown (D, double immunofluorescence for CD20 [green] and CD8 [red]). (E to G, donor MS79) Immune cell infiltrate (E, H&E staining) and CD8 T cells (F) in the meninges; double immunostaining for CD20 (green) and CD8 (red) reveals numerous CD8 T cells clustered around B cells in the meningeal infiltrate (G). (H to N, donor MS121) EBV-infected cells are visualized using EBER *in situ* hybridization; numerous EBER^+^ nuclei in the infiltrated meninges and in a perivascular cuff of a chronic WM lesion are shown (H and I, respectively). (J) Immunohistochemistry with anti-EBNA2 Ab reveals occasional perivascular cells expressing the latency III protein EBNA2 in a WM lesion. Double immunofluorescence stainings for LMP1 (green) and LMP2A or the B cell marker CD79a (red) reveal the presence of cells coexpressing LMP1 and LMP2A (K to M) and the expression of LMP1 in several perivascular CD79a^+^ B cells (N, arrows) in an active WM lesion. (O, donor MS92) Immunostaining with anti-BZLF1 MAb reveals nuclear immunoreactivity in two cells in the perivascular cuff of an active WM lesion (arrows; the inset shows the cell indicated by the arrowhead at high-power magnification). (P, donor MS92) Double immunostaining for IgA, IgG, and IgM (green) and BZLF1 (red) shows several Ig-producing plasma cells coexpressing BZLF1 in the meninges (arrows). (Q) Quantification of CD8 T cells in sections cut from 18 brain blocks of the 12 MS donors included in this study. CD8 T cells were visualized in bright field using anti-CD8 MAb and counted in the entire brain section; shown are the mean values for CD8 T cell counts in two sections of each tissue block. Nuclei were stained with hematoxylin (A, B, E, F, J, and O) or DAPI (C, D, G, K to N, and P). Bars, 200 μm (A to C and E), 100 μm (D and F to H), 50 μm (J and O), and 20 μm (I, K to N, P, and inset in O).

Fresh-frozen brain samples from 12 MS donors carrying common HLA-A (A*0201) and/or HLA-B (B*0702, B*0801) alleles (Table 1) were used to perform *in situ* stainings with MHC class I pentamers coupled to immunodominant peptides from EBV-encoded latent and lytic proteins, CMV and influenza A virus proteins as controls, and the candidate MS autoantigen myelin basic protein (MBP) (Table 2). In order to increase the chance of detecting virus-specific CD8 T cells, the brain tissue blocks analyzed in this study included immunologically active white matter (WM) lesions (active and chronic active lesions) and/or intact meninges containing substantial numbers of infiltrating CD8 T cells and B cells (Fig. 1A to G). Based on our published (19, 35–39) and unpublished data, all selected MS brain samples contained EBV-infected cells, as revealed by *in situ* hybridization for the EBV noncoding small RNA EBER (35, 37), immunohistochemistry for EBV proteins (19, 35–38), and/or real-time reverse transcription-PCR (19, 36, 39). Figure 1 shows cells expressing EBER (Fig. 1H and I), the EBV latency III protein EBNA2 (Fig. 1J), the latency II proteins LMP1 and LMP2A (Fig. 1K to M), and the EBV immediate-early lytic protein BZLF1 (Fig. 1O), as well as CD79a^+^ B cells expressing LMP1 (Fig. 1N) and Ig-producing plasma cells expressing BZLF1 (Fig. 1P), in brain samples from three of the 12 MS donors analyzed. Quantification of CD8 T cells in the MS brain sample cohort is shown in Fig. 1Q; the number of CNS-infiltrating cells stained with anti-CD8 MAb ranged between 130 and 2,200 (median value, 675) per brain section.

**TABLE 1:** HLA class I allele restriction and demographic and clinical data of MS brain tissue donors

Donor ID^*a*^	Donor HLA class I allele(s)	Sex/age at death^*b*^ (yr)	Age at onset (yr)	Disease duration (yr)	Therapy^*c*^	Cause of death^*d*^	Postmortem delay (h)
MS79	A*0201	F/49	25	24	Courses of ACTH, azathioprine, and methylprednisolone over 2 yr	MS	7
MS92	B*0801	F/37	20	17	None reported	MS	26
MS121	B*0801	F/49	35	14	Methylprednisolone for 1 yr	MS	24
MS154	B*0801	F/35	23	12	None reported	MS	12
MS180	B*0801	F/44	26	18	None reported	MS	9
MS234	B*0801	F/39	24	15	None reported	MS	15
MS286	A*0201	M/46	30	16	None reported	MS	7
MS289	A*0201, B*0702	M/45	27	18	None reported	MS	9
MS330	B*0702	F/59	20	39	None reported	MS	21
MS342	A*0201, B*0702	F/35	30	5	None reported	MS	9
MS352	B*0801	M/43	25	18	Methylprednisolone and alemtuzumab (one course each) 10 yr before death	MS	26
MS356	B*0702	F/45	29	16	None reported	MS	10

a All MS donors were in the progressive phase of the disease.

b F, female; M, male.

c ACTH, adrenocorticotropic hormone.

d MS is stated as a cause of death where death occurred as a direct result of MS or any related disability.

**TABLE 2:** HLA class I allele restriction of MS brain tissue donors and antigens and peptide epitopes analyzed by *in situ* pentamer staining

MS donor HLA class I alleles suitable for *in situ* pentamer binding and MS donor ID	EBV protein/epitope coordinates	EBV epitope sequence	CMV protein/epitope coordinates	CMV epitope sequence	Influenza A protein/epitope coordinates	Influenza A epitope sequence	MBP epitope coordinates	MBP epitope sequence
A*0201								
MS79, MS286, MS289, MS342	EBNA3C/284–293	LLDFVRFMGV	pp65/495–504	NLVPMVATV	MP/58–66	GILGFVFTL	110–118	SLSRFSWGA
	LMP1/125–133	YLLEMLWRL						
	LMP2/356–364	FLYALALLL						
	LMP2/426–434	CLGGLLTMV						
	BRLF1/109–117	YVLDHLIVV						
	BMLF1/259–267	GLCTLVAML						
	BALF4/276–284	FLDKGTYTL						
B*0702								
MS289, MS330, MS356, MS342	EBNA3A/247–255	RPPIFIRRL	pp65/265–275	RPHERNGFTVL	NP/473–481	SPIVPSFDM		
	EBNA3C/881–889	QPRAPIRPI						
	BMRF1/116–128	RPQGGSRPEFVKL						
B*0801								
MS92, MS121, MS154, MS180, MS234, MS352	EBNA3A/193–201	FLRGRAYGL	IE1/88–96	QIKVRVDMV				
	BZLF1/190–197	RAKFKQLL						

**FIG 2:**
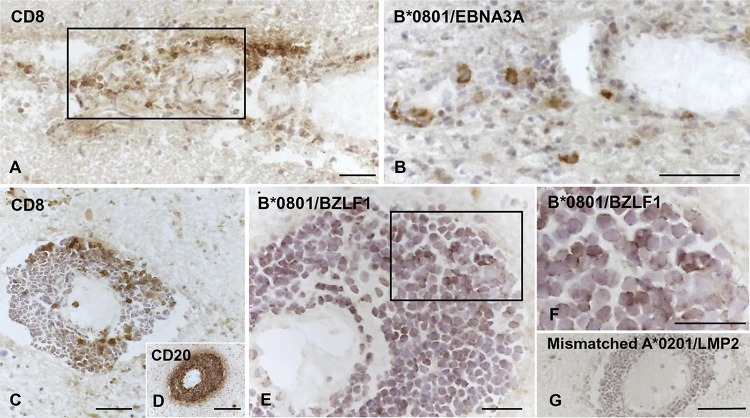
Visualization of EBV pentamer-binding cells in brain sections from HLA-B*0801^+^ MS donors. Serial brain sections from two HLA-B*0801^+^ MS donors were stained with MAbs to CD8 and CD20 and with EBV-peptide coupled pentamers. (A and B, donor MS234) Perivascular cuff containing CD8 T cells (A) and some cells binding the B*0801/EBNA3A pentamer (B) in an active WM lesion. (C to G, donor MS121) Large perivascular cuff containing scattered CD8 T cells (C), tightly packed CD20 B cells (D), and several cells binding the B*0801/BZLF1 pentamer (E; panel F shows the area within the frame in panel E at high-power magnification) at the periphery of an active WM lesion; in the same cuff, no staining is observed after incubation with HLA-mismatched A*0201/LMP2 pentamer (G). Nuclei were stained with hematoxylin. Bars, 100 μm (D and G) and 50 μm (A to C, E, and F).

In preliminary experiments, we aimed to find out whether EBV-reactive T cells could be visualized *in situ* and belonged to the CD8 T cell population. To this end, immunodominant B*0801‐restricted responses toward the EBV latent protein EBNA3A and the EBV lytic protein BZLF1 (62, 63) were investigated. Serial brain sections from four HLA B*0801^+^ MS donors were stained with anti-CD8 MAb and B*0801 pentamers coupled with EBNA3A- and BZLF1-derived peptides (Table 2). Staining of MS brain sections with HLA class I-mismatched pentamers was also performed to check the specificity of pentamer binding. Bright-field immunohistochemistry revealed that isolated or clustered EBV pentamer-binding cells were intermingled with the more numerous anti-CD8 MAb-binding cells in perivascular immune infiltrates localized within or close to active white matter lesions (Fig. 2A to F) and in the meninges (not shown). Cells binding EBNA3A or BZLF1 peptide-coupled pentamers were present in brain sections from all four HLA B*0801^+^ MS donors, while no pentamer-binding cells were observed when adjacent sections were stained with A*0201 (Fig. 2G) or B*0702 (data not shown) pentamers.

**FIG 3:**
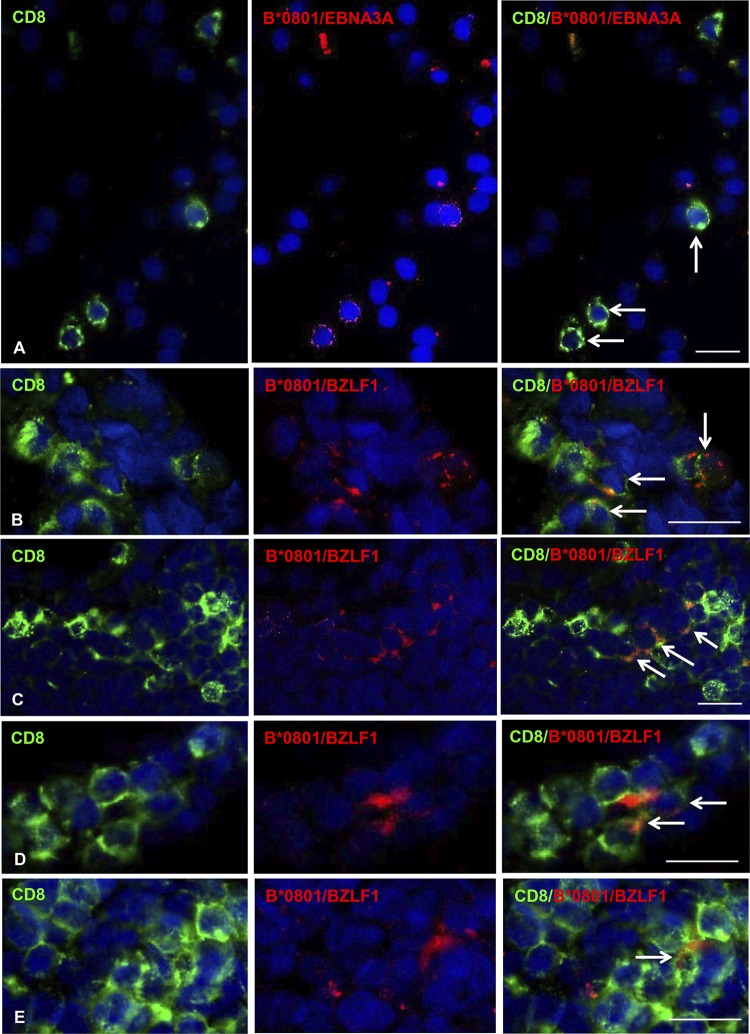
Validation of EBV pentamer binding to CD8 T cells in brain sections from HLA-B*0801^+^ MS donors. Double immunofluorescence stainings with anti-CD8 MAb (green, left column) and B*0801/EBNA3A or B*0801/BZLF1 pentamers (red, middle column) were performed in brain sections from four HLA-B*0801^+^ MS donors (MS234 [A], MS92 [B], MS121 [C], and MS180 [D and E]). Merge of CD8 and pentamer stainings (right column) reveals colocalization of CD8 immunoreactivity and pentamer binding in a subpopulation of CD8 T cells infiltrating the WM (A, C, and D) and the meninges (B and E); the arrows indicate double-labeled cells. Nuclei were stained with DAPI. Bars, 20 μm.

To validate the binding of pentamers to CD8 T cells in MS brain sections, we performed double immunofluorescence stainings with anti-CD8 MAb and B*0801/BZLF1 or B*0801/EBNA3A pentamers. As depicted in Fig. 3, binding of EBNA3A (Fig. 3A) and BZLF1 (Fig. 3B to E) pentamers was restricted to CD8 T cells in brain sections from the four B*0801^+^ MS donors analyzed, confirming the specificity and reliability of the observed stainings.

### MS brain-infiltrating EBV-specific CD8 T cells recognize viral proteins expressed in different phases of viral infection.

We next searched and quantified EBV-reactive CD8 T cells in all brain samples included in this study (18 brain tissue blocks from 12 MS donors) using HLA-A*0201, B*0702, and B*0801 pentamers conjugated with identified immunodominant peptides from nine viral proteins expressed in different phases of the EBV life cycle: EBNA3A and EBNA3C (latency III or growth program), LMP1 and LMP2 (latency III and latency II programs), BZLF1 and BRLF1 (immediate-early lytic cycle), BMLF1 and BMRF1 (early lytic cycle), and BALF4 (late lytic cycle) (Table 2). Cells binding EBV pentamers were visualized in bright field in brain sections from 11 MS donors (16 tissue blocks); two brain samples from one HLA-A*0201^+^ donor (MS286) did not bind any of 5 different A*0201/EBV peptide-coupled pentamers or any other control pentamer (Table 3). These samples were among the less infiltrated (Fig. 1Q) and were excluded from the statistical analysis.

**TABLE 3:** Percentages of CD8 T cells recognizing EBV, CMV, influenza A virus, and MBP protein-derived peptides in the total CD8 T-cell population infiltrating the MS brain

Donor ID and HLA class I allele	Brain tissue block analyzed	% of pentamer-binding cells in the total CD8 population															
		EBV											CMV		Influenza A virus		MBP
		EBNA3A	EBNA3C	LMP1	LMP2	BZLF1	BRLF1	BMRF1	BMLF1	BALF4	Sum of % of EBV pentamer- binding cells^*a*^	EBNA3A + BZLF1^*b*^	pp65	IE1	MP	NP	
MS79																	
A*0201	A3B5			0.9	1.0				1.7		3.6		0.3		0		0
	A1D5		1.3	0.8	1.1		0.8		1.7		5.7						
	A1B4			2.7	1.8		1.7		1.8	0.7	8.7						
MS286																	
A*0201	A1A3				0				0	0	0		0		0		0
	A1C1		0	0	0					0	0						
MS330																	
B*0702	A6A1	1.9	1.0					0.6			3.5		0			0	
MS356																	
B*0702	A3B4	1.0	0.8					0			1.8		0			0	
MS289																	
A*0201	A1A2		1.7	1.7	0		1.7		4.1	0	19.2		0		0		0
B*0702		2.0	3.5					4.5					0			0	
A*0201	P2B7		NA^*c*^	1.8	0		1.3		3.8	1.6	12.5		0		0		0
B*0702		NA	0.9					3.1									
MS342																	
A*0201	P1C1		0.3	1	0.8		0		0.4	0.5	3.6		0		0		0
B*0702		0.2	0.2					0.2								0	
MS92																	
B*0801	A5B2	0.5				2.2					2.7			0.2			
MS121																	
B*0801	P3D4	0.2				2.7					2.9			0			
MS154																	
B*0801	A2E3	0				3.8					3.8	3.8		0			
MS180																	
B*0801	P1B4	2.0				3.1					5.1			0			
	A1D7	0.4				1					1.4			0			
MS234																	
B*0801	P4C3	1.6				3.5					5.1	9.0		0.2			
MS352																	
B*0801	P1B5	1.8				1.3					3.1	7.7		0.2			
	P5C3	0.1				1.8					1.9			0			

a The figures in this column were obtained by summing the percentages of cells binding different EBV protein-derived peptides in serial sections from the same brain tissue block.

b The figures in this column represent the percentages of pentamer-binding cells in brain sections incubated simultaneously with EBNA3A/B*0801 and BZLF1/B*0801 pentamers.

c NA, not assessable.

**FIG 4:**
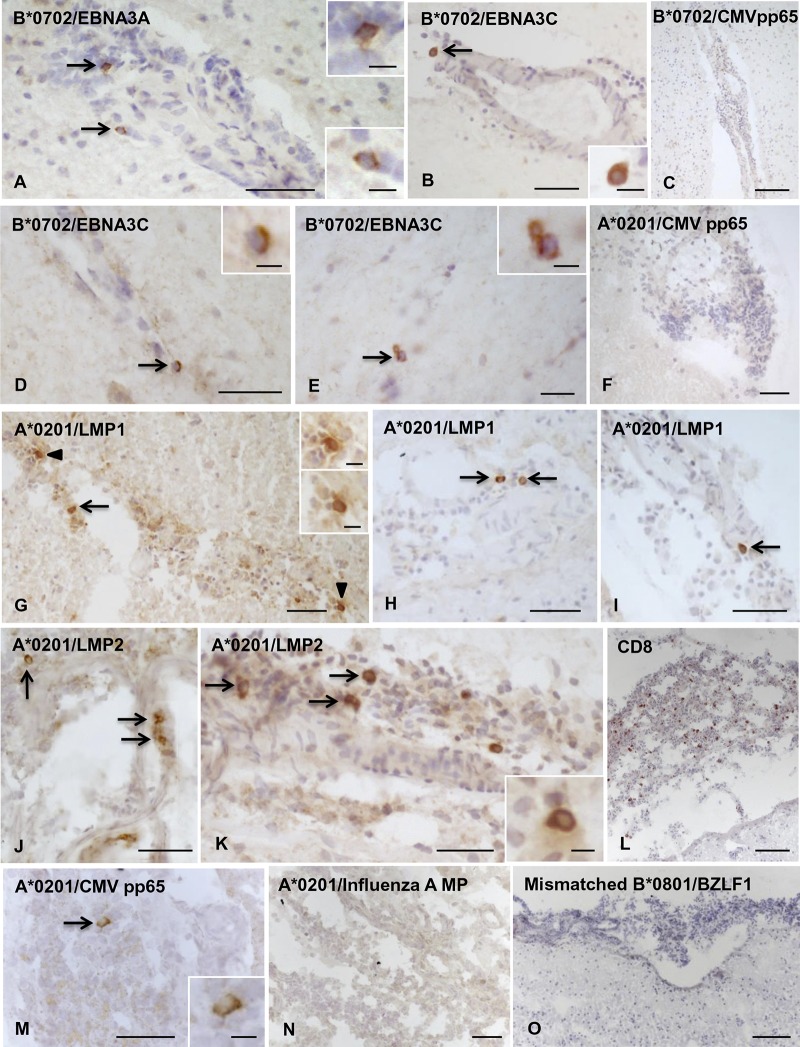
Localization of CD8 T cells specific for EBV latent proteins in MS brain sections. Cells binding pentamers coupled with peptides from different EBV latent proteins were visualized in bright field in brain sections from HLA-A*0201^+^, B*0702^+^, and B*0801^+^ MS donors. (A to C, donor MS356, B*0702^+^) Presence of CD8 T cells specific for B*0702-restricted EBNA3A (A) and EBNA3C (B) peptides, but not for B*0702-restricted CMV pp65 peptide (C), in the inflamed meninges; the insets show the cells indicated by the arrows in panels A and B at high-power magnification. (D to F, donor MS289, A*0201^+^/B*0702^+^) Perivascular CD8 T cells specific for B*0702-restricted EBNA3C peptide are indicated by the arrows in panels D and E and shown at high-power magnification in the insets. (F) Absence of CD8 T cells specific for A*0201-restricted CMV pp65 peptide in a meningeal infiltrate. (G to O, donor MS79, A*0201^+^) Presence of CD8 T cells specific for A*0201-restricted LMP1 peptide (G to I, arrows; the insets in panel G show the cells indicated by the arrowheads at high-power magnification) and LMP2 peptide (J and K, arrows) in the meninges where numerous scattered CD8 T cells are also detected (L). In adjacent sections, one CD8 T cell specific for A*0201-restricted CMV pp65 peptide (M; the inset shows the cell indicated by the arrow at high-power magnification) but no CD8 T cells specific for A*0201-restricted influenza A MP peptide (N) or cells binding B*0801/BZLF1 pentamer (O) were detected. Nuclei were stained with hematoxylin. Bars, 100 μm (C, L, and O), 50 μm (A, B, D, F to K, M, and N), 20 μm (E and inset in panel M), and 10 μm (insets in panels A, B, D, E, G, and K).

**FIG 5:**
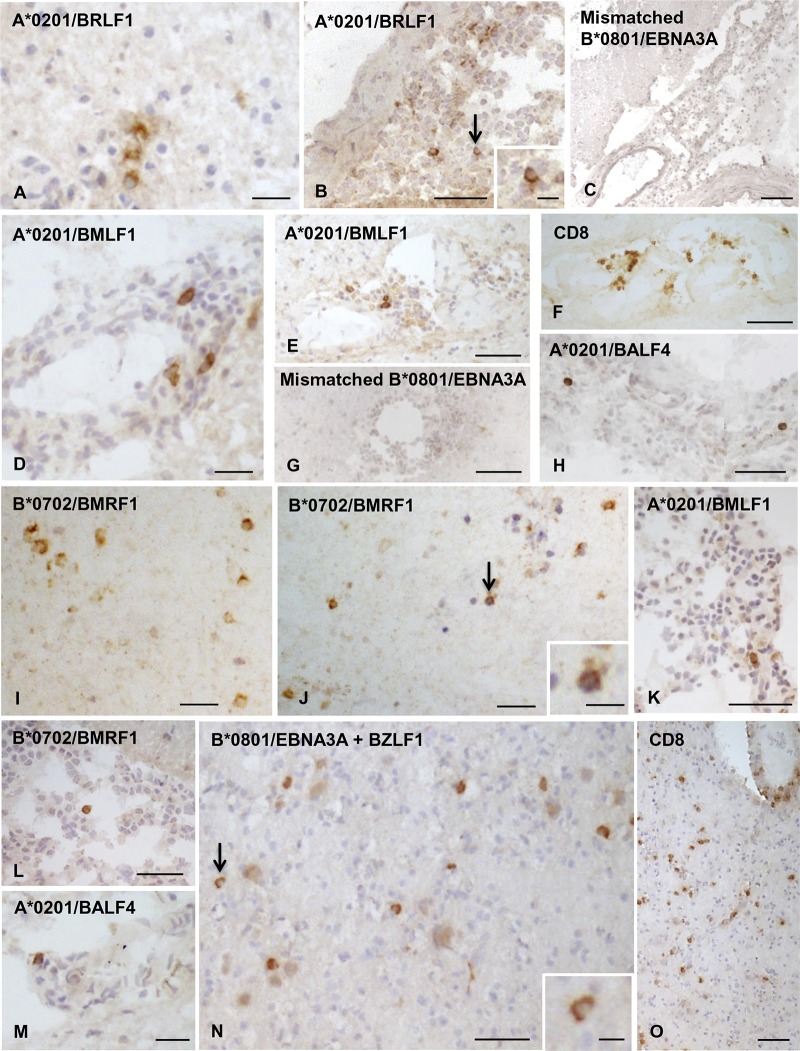
Localization of CD8 T cells recognizing EBV lytic proteins in MS brain sections. Cells binding pentamers coupled with peptides from different EBV lytic proteins were visualized in bright field in brain sections from HLA-A*0201^+^, B*0702^+^, and B*0801^+^ MS donors. (A to H, donor MS79, A*0201^+^) CD8 T cells specific for A*0201-restricted BRLF1 peptide in WM (A) and meninges (B; the inset shows the cell indicated by the arrow at high-power magnification) but no staining in the meninges with mismatched B*0801/EBNA3A pentamer (C). Perivascular CD8 T cells specific for A*0201-restricted BMLF1 peptide (D and E) among infiltrating CD8 T cells (F) but no cells binding mismatched B*0801/EBNA3A pentamer (G) in a WM lesion. (H) Isolated CD8 T cells specific for A*0201-restricted BALF4 peptide in the meninges. (I to K, donor MS289, A*0201^+^/B*0702^+^) Several CD8 T cells specific for B*0702-restricted BMRF1 peptide scattered in an active WM lesion (I and J; the inset in panel J shows the cell indicated by the arrow at high-power magnification) and one CD8 T cell specific for A*0201-restricted BMLF1 peptide in the meninges (K). (L and M, donor MS342, A*0201+/B*0702^+^) Isolated CD8 T cells specific for B*0702-restricted BMRF1 peptide (L) and A*0201-restricted BALF4 peptide (M) in the meninges. (N and O) (MS234, B*0801^+^) Incubation of brain sections with pooled B*0801/EBNA3A and B*0801/BZLF1 pentamers reveals several pentamer-binding cells scattered throughout an active WM lesion (N; the inset shows the cell indicated by the arrow at high-power magnification); staining of a serial section with anti-CD8 MAb shows widespread CD8 T cell infiltration of the same WM lesion. Nuclei were stained with hematoxylin in panels A to E, H, and J to O. Bars, 100 μm (C and O), 50 μm (B, E to H, K, L, and N), 20 μm (A, D, I, J, M, and inset in panel B), and 10 μm (inset in panels J and N).

In the sample cohort displaying EBV pentamer binding, CD8 T cells to individual EBV peptides were visualized in all or the majority of tissue blocks analyzed (median, 100%; range, 67 to 100%). The CD8 T cells specific for EBV latent and lytic proteins (Fig. 4 and 5, respectively) were observed in the infiltrated meninges and/or within perivascular cuffs of small to large size in active WM lesions and at the active edge of chronic WM lesions. In two donors (MS234 and MS289), both CD8-expressing cells and EBV-specific CD8 T cells were also found scattered throughout active WM lesions (Fig. 5I, J, N, and O).

**FIG 6:**
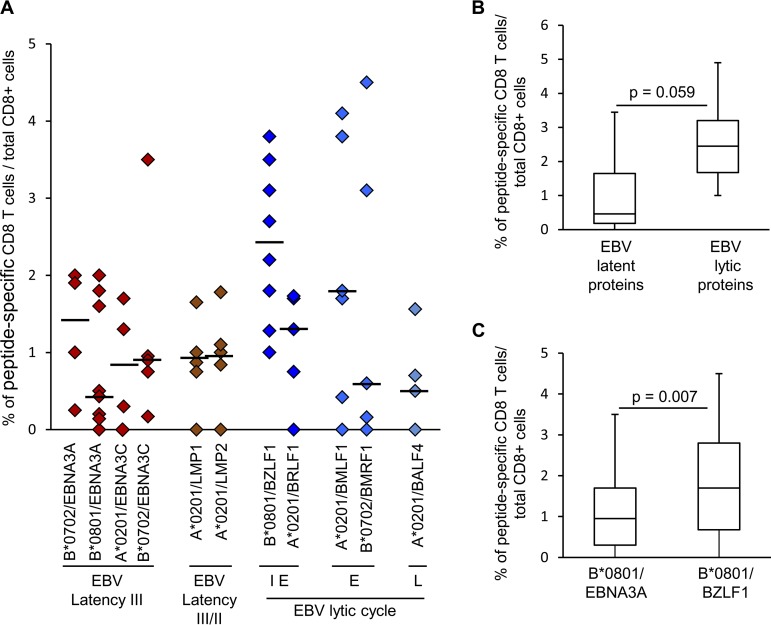
Frequency of T cells specific for EBV proteins in the MS brain. Data are expressed as percentages of CD8 T cells specific for individual immunodominant peptides in the total CD8 population. (A) Each symbol represents the frequency of cells binding a given pentamer in each tissue block analyzed (*n* = 18 from 12 MS donors); the line marks the median value. Different phases of the EBV lytic cycle are indicated: IE, immediate early; E, early; L, late. (B) Comparison of the frequencies of CD8 T cells specific for EBV latent and lytic proteins assessed in 16 brain tissue blocks from 11 MS donors. (C) Comparison of the frequencies of CD8 T cells recognizing B*0801-restricted BZLF1 and EBNA3A peptides assessed in 8 tissue blocks from 6 HLA-B*0801^+^ MS donors. In panels B and C, *P* values were assessed by Mann-Whitney test. The lines inside the boxes represent the median values; boxes extend from the 25th to the 75th percentile, covering the interquartile range (IQR), and whiskers extend from the 25th percentile, −1.5 IQR, to the 75th percentile, +1.5 IQR.

The frequency of CD8 T cells recognizing individual EBV protein-derived peptides was calculated by dividing the number of pentamer-binding cells counted in the entire brain section by the number of cells stained with anti-CD8 MAb in an adjacent section. For each EBV peptide analyzed, there was high variability in the frequency of pentamer-binding cells visualized in brain sections from different donors or from different brain tissue blocks of the same donor (Table 3 and Fig. 6A). The percentage (median value) of EBV peptide-specific CD8 T cells ranged between 0.5 and 2.5% of the total CD8 cell population (Fig. 6A). Frequencies above 3% (up to 4.5%) were found only in brain sections stained with pentamers coupled with peptides from the latency III protein EBNA3C, the immediate-early protein BZLF1, and the early lytic proteins BMLF1 and BMRF1 (Table 3 and Fig. 6A). Using the Kruskal-Wallis test, no significant differences were found in the frequency of CD8 T cells recognizing EBV latency or lytic cycle-related proteins. Collectively, however, CD8 T cells recognizing EBV lytic antigens tended to be more frequent than those recognizing EBV latent proteins (Fig. 6B). Only in the HLA-B*0801^+^ sample subgroup (8 brain samples from 6 MS donors) was the frequency of CD8 T cells recognizing BZLF1 significantly higher than that of CD8 T cells recognizing EBNA3A (Fig. 6C).

The frequency of EBV-specific CD8 T cells raised to 12 to 19% when the frequencies of CD8 T cells reactive to different EBV peptides in serial brain sections from the same tissue block were summed and to 8 to 9% when brain sections were incubated with a mixture of two EBV pentamers (i.e., B*0801/EBNA3A and B*0801/BZLF1) (Table 3 and Fig. 5N). These data suggest that EBV-specific CD8 T cells account for a substantial proportion of CNS-infiltrating CD8 T cells.

### CD8 T cells recognizing common viruses and MBP are less frequent than EBV-reactive CD8 T cells or are undetectable in the MS brain.

**FIG 7:**
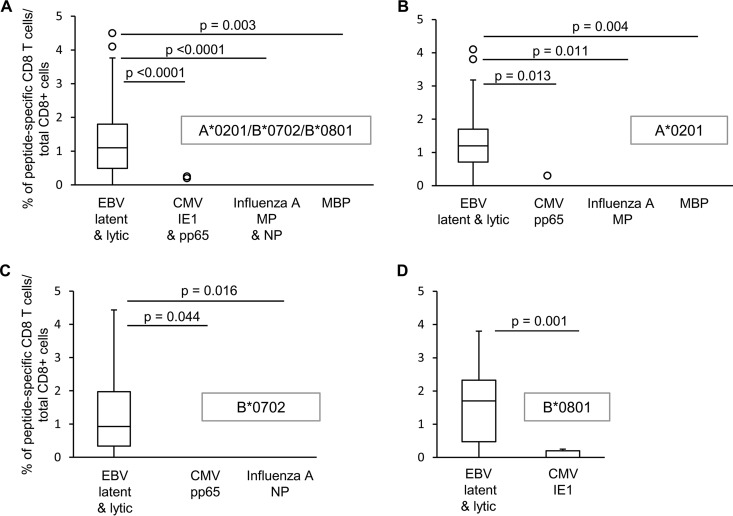
Comparison of the frequencies of EBV-, CMV-, influenza A virus-, and MBP-specific CD8 T cells infiltrating the MS brain. The frequencies of CD8 T cells specific for viral protein- and MBP-derived peptides were compared irrespective of the HLA background (A) and in the same HLA background (A*0201 [B], B*0702 [C], or B*0801 [D]). Data are expressed as percentages of peptide-specific CD8 T cells in the total CNS-infiltrating CD8 population. *P* values were assessed by Kruskal-Wallis test with Dunn correction. The lines inside the boxes represent the median values; boxes extend from the 25th to the 75th percentile, covering the interquartile range (IQR), and whiskers extend from the 25th percentile, −1.5 IQR, to the 75th percentile, +1.5 IQR. Maximum outliers outside the whiskers are represented by individual marks.

We then asked whether CD8 T cells specific for immunodominant proteins of other common viruses, like CMV and influenza A virus, or the putative CNS autoantigen MBP could be visualized in the MS brain and if their frequency differed from that of EBV-specific T cells. CD8 T cells recognizing CMV were searched in brain samples from 12 MS donors using HLA-A*0201 and HLA-B*0702 pentamers conjugated with two different peptides from pp65, the major CMV structural protein, and HLA-B*0801 pentamers conjugated with a peptide from IE1, a major CMV immediate-early protein (Table 2). CMV-specific CD8 T cells were detected in brain sections from 4 of 12 MS donors (Fig. 4C, F, and M), accounting for 0.2 to 0.3% of the total CD8 population (Table 3). No influenza A virus-specific CD8 T cells were detected in brain sections from 6 MS donors (Table 3 and Fig. 4N) when HLA-A*0201 and HLA-B*0702 pentamers coupled to influenza A virus matrix protein (MP)- and nucleoprotein (NP)-derived peptides, respectively, were used (Table 2). In addition, no MBP-specific CD8 T cells were detected in brain samples from 4 HLA-A*0201^+^ MS donors (Table 3). The frequencies of CNS-infiltrating CD8 T cells recognizing CMV, influenza A virus, or MBP-derived peptides were always significantly lower than the frequencies of CD8 T cells recognizing EBV protein-derived peptides, both through all-HLA backgrounds (Fig. 7A) and by comparing the response to A*0201-, B*0702-, or B*0801-restricted epitopes (Fig. 7B to D).

### EBV-reactive CD8 T cells exhibit cytotoxic activity and contact EBV-infected cells in the MS brain.

**FIG 8:**
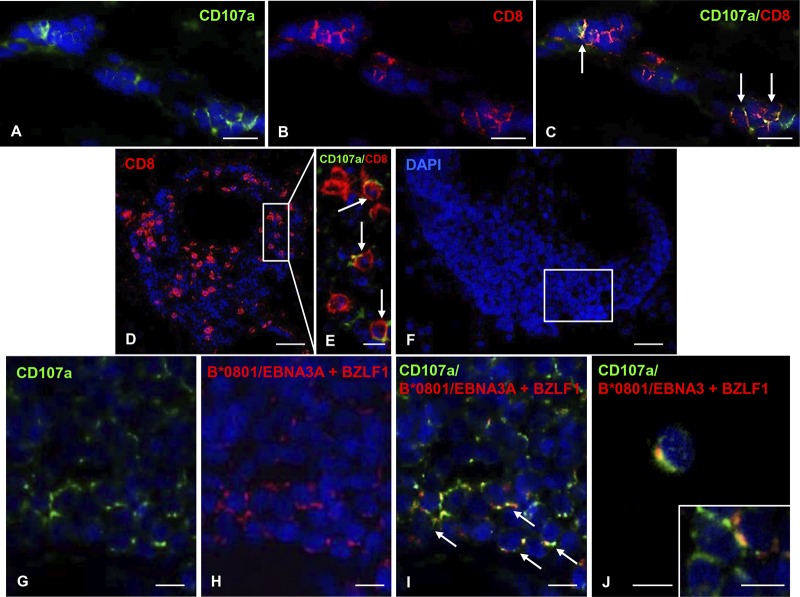
Cytotoxic activity of MS brain-infiltrating EBV-specific CD8 T cells. Double immunofluorescence staining of MS brain sections with MAb to the degranulation marker CD107a (A, green) and polyclonal Ab to CD8 (B, red) reveals the presence of a subpopulation of CD107a^+^/CD8^+^ cells among perivascular CD8^+^ T cells in an active WM lesion of a B*0801^+^ MS180 donor (C, arrows). (D and E) CD8 T cells (D), some of which coexpress CD107a (E, arrows; the area marked by the frame in panel D is shown at high-power magnification) in a large perivascular cuff, in an active WM lesion of the B*0801^+^ MS121 donor. (F) DAPI nuclear staining of a large perivascular cuff in an active WM lesion of the B*0801^+^ MS121 donor. (G to I) Double immunofluorescence staining with anti-CD107a MAb (G, green) and pooled B*0801/EBNA3A and B*0801/BZLF1 pentamers (H, red) reveals presence of CD107a^+^ pentamer-binding cells (I, arrows) in an area of the perivascular cuff shown in panel F. Two CD107a^+^ pentamer-binding cells in a different area of the same perivascular cuff are shown in panels J and the inset. Serial sections were used for the stainings shown in panels F to J and in Fig. 2C to F; note that in Fig. 2E and F several BZLF1 pentamer-binding cells are visualized in bright field in the same B-cell-rich cuff. Nuclei were stained with DAPI. Bars, 50 μm (D), 20 μm (A to C and F), and 10 μm (E, G to J, and inset in panel J).

Since polyfunctional CD8 T cells producing multiple inflammatory cytokines and displaying cytotoxic activity are extremely effective in controlling viral infections, we sought to gain insights into the effector functions of CNS infiltrating EBV-specific CD8 T cells. In an initial set of experiments, brain sections from HLA-B*0801^+^ MS donors were simultaneously labeled with EBV peptide-coupled pentamers (a mixture of EBNA3A/B*0801 and BZLF1/B*0801 pentamers) and antibodies to the proinflammatory cytokine gamma interferon (IFN-γ) or the lytic enzyme granzyme B, both of which are expressed by CD8 T cells infiltrating the MS brain (19, 35, 38, 53, 58, 59). However, neither of these intracellular antigens could be visualized by immunofluorescence in fresh-frozen brain tissue (data not shown). Hence, to evaluate the cytotoxic function of EBV pentamer-binding cells, we performed double immunofluorescence stainings with the above-mentioned pentamers and a MAb specific for the degranulation marker CD107a. In agreement with previous data (35, 59), membrane expression of CD107a, a component of cytotoxic granules exposed on the cell surface after release of lytic granule contents, was observed in a proportion (approximately 15%; range, 7 to 20%) of CNS-infiltrating CD8 T cells (Fig. 8A to E). Furthermore, membrane CD107a staining was observed in virtually all cells binding pooled HLA-B*0801/EBNA-3A and HLA-B*0801/BZLF1 pentamers (Fig. 8G to J), indicating ongoing cytotoxic activity of EBV-specific CD8 T cells recognizing two highly immunodominant EBV antigens (62, 63).

**FIG 9:**
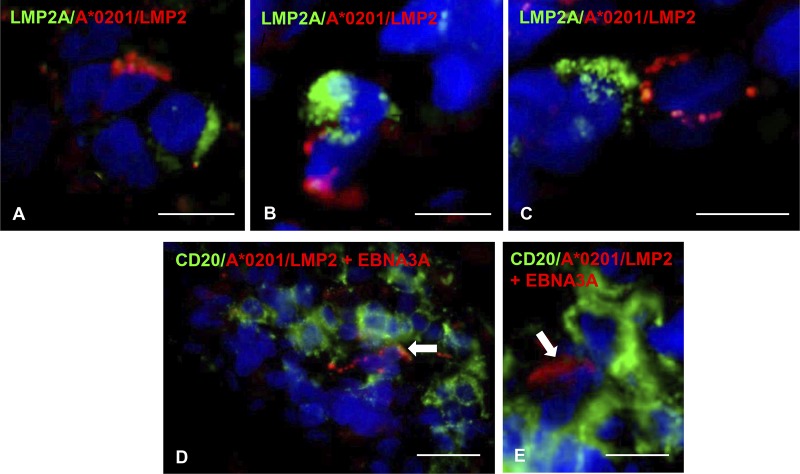
EBV-specific CD8 T cells contact EBV-infected cells in the MS brain. (A to C) Double immunofluorescence staining of brain sections from the A*0201^+^ MS79 donor with MAb specific for the EBV latent protein LMP2A (green) and pooled A*0201 pentamers coupled with LMP2 426–434 and LMP2 356–364 peptides (red) reveals the presence of tight contacts between LMP2A-expressing cells and pentamer-binding cells in the meninges. (D and E) Double immunofluorescence staining of a brain section from the same donor with anti-CD20 MAb (green) and pooled A*0201 pentamers coupled with LMP2 426–434, LMP2 356–364, and EBNA3C peptides (red) shows individual pentamer-binding cells in contact with B cells within a B-cell-rich meningeal infiltrate. Nuclei were stained with DAPI. Bars, 20 μm (D) and 10 μm (A to C and E).

We next asked whether EBV-specific CD8 T cells interact directly with EBV-infected cells in the MS brain. Due to technical reasons (i.e., incompatibility of tissue fixation and/or use of rabbit polyclonal antibodies to EBV antigens with the pentamer staining procedure), the only EBV antigen that could be analyzed in conjunction with the corresponding pentamer was the latent protein LMP2A. Staining of brain sections from one HLA-A*0201 MS donor (MS79) with antibodies to LMP2A and a mixture of A*0201/LMP2 356–364 and A*0201/LMP2 426–434 pentamers allowed us to visualize EBV-specific CD8 T cells contacting or juxtaposed to LMP2A^+^ cells within the perivascular space of inflamed blood vessels in white matter lesions and in the meninges (Fig. 9A to C). Staining of brain sections from the same A*0201^+^ MS donor with pooled A*0201/LMP2 356–364, A*0201/LMP2 426–434, and A*0201/EBNA3C pentamers and anti-CD20 MAb also allowed us to visualize EBV-specific CD8 T cells contacting B cells (Fig. 9D and E). These observations suggest that encounter between EBV-specific CD8 T cells and infected B cells expressing the cognate viral antigen occurs in the MS brain, possibly leading to T-cell activation and killing of virus-infected cells.

## DISCUSSION

The aim of this study was to test the hypothesis that EBV-specific CD8 T cells are involved in CNS tissue damage in MS by mediating an immunopathological response toward a persistent intracerebral EBV infection that was documented in previous studies performed in our (19, 35–39) and other (40–42) laboratories.

The key finding is that EBV-specific CD8 T cells are commonly found in the MS brain and are significantly more frequent than CMV-specific CD8 T cells. Furthermore, CD8 T cells recognizing influenza A virus or a putative CNS autoantigen are not detected. These data suggest that entry of EBV-specific CD8 T cells into the MS brain results from active recruitment rather than nonspecific extravasation due to the local inflammatory process. The findings obtained in MS brain tissue are in line with previous studies showing that CD8 T cells recognizing EBV, but not CMV or several candidate CNS autoantigens, accumulate in the CSF of MS patients (54–57). In a recent study performed in postmortem MS brain tissue (53), T cells, mainly CD8 T cells, recovered from WM lesions did not recognize nine candidate CNS autoantigens, including MBP, but did recognize autologous EBV‑transformed B-cell lines; however, the cognate antigen, EBV or a B-cell-associated antigen, was not identified. Taken together with neuropathological evidence of active EBV infection in CNS-infiltrating B cells/plasma cells (19, 35–39, 42) and the predominance of CD8 T cells with a cytotoxic effector phenotype in the MS brain (50–53), the selective enrichment of EBV-specific CD8 T cells in postmortem MS brain samples reported here and in the CSF of MS patients (54–57) supports a pathogenic model of MS where skewed cytotoxic immune responses toward EBV may contribute to inflammation in the CNS. Failure to eradicate a chronic active EBV infection in the MS brain should lead to a vicious circle of viral antigens stimulating the anti-EBV immune response, which maintains local inflammation with devastating effects in a tissue with very limited regenerative capacity. In the context of chronic immune stimulation, a few EBV-specific CD8 T cells may be sufficient to amplify inflammation by activating macrophages and recruiting more antigen-specific as well as bystander T cells. This disease model is consistent with the notion that CD8 T cells are the main drivers of bystander tissue damage in EBV-associated immunopathologic diseases (7, 49). It also shows analogies with the pathogenesis of another inflammatory demyelinating disease of the CNS, human T-lymphotropic virus type 1 (HTLV-1)-associated myelopathy (HAM) or tropical spastic paraparesis, which is an infrequent complication of HTLV-1 infection (64). In HAM, circulating HTLV-1-infected T cells invade the CNS and trigger an immunopathologic response against the virus which damages neural cells (64). By analogy, MS could be considered a rare neurological complication of the most common EBV infection (4).

An EBV-centered pathogenic model of MS could also explain why B-cell-depleting therapy, which is used in EBV-associated lymphoproliferative disease to eliminate EBV-transformed B cells, is highly effective in MS (24, 25, 32). In this disease, depletion of B cells by anti-CD20 MAbs would lower the viral burden and, consequently, the CNS-directed immunopathological response. Recently, we described profound EBV deregulation in the CNS and deep cervical lymph node, but not pulmonary lymph node, of a patient with primary progressive MS (65). We also observed a prominent proliferation of CD20-negative immunoblasts in the cervical lymph node paracortex, a finding indicative of T-cell activation (65). Although preliminary, these findings point to CNS-draining lymph nodes as the key site where an immunopathological response targeting intracerebral EBV is stimulated. Depletion of B cells supporting an active EBV infection in CNS-draining lymph nodes could explain the rapid therapeutic effect of anti-CD20 MAbs in MS despite low penetration in the CNS (66). The finding that rituximab causes depletion of B cells and a marked decline of T cells in the CSF of treated MS patients further suggests that EBV-infected B cells sustain pathogenic T cell responses (67, 68).

The second main finding of this study is that EBV antigen recognition by CNS-infiltrating CD8 T cells encompasses a wide range of proteins expressed in different phases of the EBV life cycle. These include nuclear (EBNA3A and EBNA3C) and membrane proteins (LMP1 and LMP2) expressed during EBV latency and BZLF1, BRLF1, BMLF1, BMRF1, and BALF4 proteins expressed during immediate-early, early, and late phases of the viral lytic cycle (6, 7). This finding is consistent with detection of a similarly wide range of EBV proteins and/or transcripts in postmortem MS brain samples, including EBNA2, EBNA3A, LMP1, LMP2A, BZLF1, BFRF1, BMRF1, gp350/220, and p160 (19, 35, 36, 38, 39, 42, 58, 59). It should be stressed here that, analogous to the present study, EBV proteins and/or transcripts were detected in almost all MS brains analyzed when the samples included B-cell-containing immune infiltrates. The consistency of these findings corroborates the concept that intracerebral EBV infection and the CD8 T cell-mediated response to EBV play a role in MS pathogenesis.

A well-established hierarchy exists among CD8 T-cell responses that target EBV antigens. In particular, EBV latency III antigens EBNA3A/3B/3C are immunodominant, whereas the EBV latency II antigens, EBNA1/LMP1/LMP2A, are subdominant (62). EBV lytic cycle antigens also display a hierarchy of immunodominance, as the two immediate-early antigens, BRLF1 and BZLF1, and some early antigens elicit stronger responses than late lytic antigens (62, 63). In this study, no preferential CNS infiltration by CD8 T cells specific for the most immunodominant EBV protein-derived peptides was observed. However, CD8 T cells specific for EBV lytic proteins tended to be more frequent than those specific for EBV latent proteins, and CD8 T cells recognizing BZLF1 were significantly more frequent than those recognizing EBNA3A in the HLA-B*0801 background. Of interest, the frequency of CD8 T cells specific for EBV lytic antigens, including BZLF1, in the peripheral blood of patients with relapsing MS was higher during disease flares, while the CD8 T-cell response to EBV latent antigens increased during remissions (19). Since the MS brain samples analyzed in this study were characterized by a high degree of immune cell infiltration, it is plausible that more pronounced CNS inflammation is associated with a relatively higher frequency of EBV lytic antigen-specific CD8 T cells in brain tissue.

The third main finding of this study is that EBV-specific CD8 T cells recruited to the MS brain have a cytotoxic phenotype and contact EBV-infected cells. Cytotoxic activity revealed by perforin, granzyme B, and membrane CD107a immunostainings, as well as tight contacts with EBV-infected cells, was previously demonstrated for MS brain-infiltrating CD8 T cells (19, 35, 38, 58, 59). For technical reasons, in this study only expression of membrane CD107a in the subpopulation of EBV-specific CD8 T cells and contacts between EBV latently infected cells expressing LMP2A- and LMP2-specific CD8 T cells were investigated. The results suggest that EBV-specific CD8 T cells infiltrating the MS brain become activated after recognition of their cognate antigen on EBV-infected cells and kill their target cells. However, it is likely that not all encounters between CNS-infiltrating EBV-specific CD8 T cells and EBV-infected cells lead to elimination of the latter. Because EBV-encoded proteins and viral microRNAs have immune evasion functions (69, 70), the cytotoxic response could fail to fully control intracerebral EBV infection, thereby perpetuating inflammation. It is also known that the T-cell-inhibitory molecule PD-L1 is upregulated by EBV latent proteins and may inhibit the immune response to EBV^+^ tumors (71–73). Further experiments are needed to establish if PD-L1 is expressed on EBV-infected B cells in the MS brain and plays a role in suppressing the local activation of PD-1^+^ cytotoxic T cells (53, 74).

If EBV is the main antigenic stimulus promoting immune-mediated inflammation in MS, it should be possible to treat MS by normalizing the EBV-host balance with antiviral drugs or T-cell therapy. Previous trials with antiherpesvirus drugs in MS did not show any significant improvement in clinical and radiological parameters but highlighted a possible effect in a subgroup with high disease activity (75). Interestingly, a recent study described the case of an HIV-negative patient with relapsing-remitting MS who achieved significant and sustained disease improvement upon treatment with zidovudine/lamivudine (Combivir), a highly active antiretroviral therapy (76). It was suggested that the beneficial response to zidovudine/lamivudine is related to the action of zidovudine, a known inhibitor of EBV lytic DNA replication (77). Based on the assumption that defective CD8^+^ T-cell immunity to EBV leads to CNS colonization by EBV-infected autoreactive B cells (21, 22), adoptive immunotherapy with *in vitro*-expanded autologous T cells directed against EBV latent proteins was recently used to treat MS patients (78). In a phase I trial in progressive MS, EBV-specific immunotherapy had no adverse effects, but the therapeutic efficacy of this treatment has to be evaluated in larger clinical trials (78).

The association between MS and the B-lymphotropic EBV is one of the best documented pathogen-chronic disease associations (4, 5, 10, 11), leading to the possibility that prevention or reduction of the risk to develop MS could be achieved with EBV vaccines (79, 80). To date, any theory of MS pathogenesis must explain the following, undisputed observations: increased MS risk after infectious mononucleosis; elevated immune reactivity to EBV, but not to any other tested pathogen; CD8 T cell predominance in the CNS inflammatory infiltrates; and high therapeutic efficacy of B-cell-depleting anti-CD20 MAbs. The present study offers a possible explanation for these phenomena and a rationale for current and future treatments for MS.

## MATERIALS AND METHODS

### Brain tissues and sample selection.

Postmortem frozen tissue blocks (about 4 cm^3^ each) from the cerebral hemispheres of persons with MS were obtained from the UK MS Society Tissue Bank at Imperial College London. Use of postmortem human tissues for research purposes was approved by the Ethics Committee of the ISS. Information on donors’ HLA class I alleles was obtained through the UK Multiple Sclerosis Tissue Bank for samples included in a previous MS genome-wide association study (81). For 11 donors, HLA genotypes were identified by HLA sequencing-based typing (Abbott Molecular) of brain-derived DNA; genomic DNA was extracted from MS brain tissue (15 to 20 mg) using a QIAamp DNA minikit (Qiagen, Valencia, CA) by following the manufacturer’s instructions.

Thirty-six snap-frozen brain tissue blocks from 24 MS cases who died in the progressive phase of MS and carried MHC class I alleles, enabling analysis of *in situ* pentamer binding using commercially available pentamers (Pro5 MHC class I pentamers; ProImmune, Oxford, UK), were included in a preliminary screening to select samples with good tissue preservation and substantial immune cell infiltration. For neuropathological assessment, brain sections (10 μm) were stained as described previously (19, 35, 36, 38). The extents of demyelination and lesion inflammatory activity were evaluated by immunostaining for myelin-oligodendrocyte glycoprotein (MOG) (kind gift of S. Piddlesden, Cardiff, UK) and MHC class II molecules, respectively (19, 35, 36). The degree of leukocyte infiltration in the meninges and white matter was evaluated using hematoxylin and eosin (H&E) staining and by immunohistochemistry using antibodies to B-cell (CD20 and CD79a), plasma cell (IgA, IgG, and IgM), and T-cell (CD8) markers (19, 35, 36, 38). The presence of degranulating CD8 T cells was assessed by double immunofluorescence for CD8 and CD107a, as described previously (35). After extensive neuropathological characterization, 18 brain blocks from 12 MS donors were selected for this study (Table 1). Based on the available clinical documentation, no immunotherapy was reported in the 6 months before death. HLA class I allele restriction, demographic and clinical data of the MS donors included in this study, and postmortem delay of brain tissue collection are shown in Table 1.

### EBV detection.

EBER *in situ* hybridization was performed using the PNA probe, which hybridizes with both EBER1 and EBER2, and the detection kit from DakoCytomation (Glostrup, Denmark), as described previously (35, 37).

Immunohistochemical stainings for EBV latent (EBNA2) and lytic (BZLF1) proteins and double immunofluorescence stainings for LMP1 and the B-cell marker CD79a and for BZLF1 and the plasma cell marker IgA, IgG, and IgM were performed in sections from paraformaldehyde (PFA)-fixed frozen brain tissue blocks of three MS donors analyzed in this study (MS79, MS92, and MS121), as previously described (19, 35–38). For double immunofluorescence staining for LMP1 and LMP2A, brain sections were incubated with anti-LMP2A rat MAb (1:50; clone TP4E11; Ascenion, Munich, Germany) and anti-LMP1 mouse MAb (1:100; clone CS.1-4; DakoCytomation) in phosphate-buffered saline (PBS) containing 1% bovine serum albumin overnight at 4°C, and, after washing with a mixture of Alexa Fluor 488-conjugated donkey anti-mouse IgG (Invitrogen, Eugene, OR) and tetramethylrhodamine (TRITC)-conjugated donkey anti-rat Ig (Jackson ImmunoResearch Laboratories, Cambridgeshire, UK), both diluted 1:300 in PBS containing 3% normal donkey serum. After further washings, sections were mounted with antifade mounting medium containing 4′,6-diamidino-2-phenylindole (DAPI) (Invitrogen).

### *In situ* pentamer staining.

Each snap-frozen brain tissue block was cut in series of twenty 10-μm sections with a cryostat; sections were air dried for 12 h at room temperature (RT) and stored at –80°C for no longer than 4 weeks. The first, the tenth, and the last sections of each consecutive series were stained with anti-CD8 MAb (DakoCytomation) and were used for quantification of CD8 T cells. The other sections were used to investigate pentamer binding. *In situ* pentamer stainings were performed in bright field using R-phycoerythrin (R-PE)-labeled Pro5 MHC class I pentamers (ProImmune) by following the manufacturer’s instructions, with minor modifications. Air-dried, frozen brain sections were maintained at RT for 90 min, rehydrated with 3 washes (2 min each) in PBS, and incubated with 10% normal goat serum (NGS) (Sigma-Aldrich, St. Louis, MO) for 30 min. Sections were then incubated overnight at 4°C with HLA class I pentamers (diluted 1:5 in PBS, final concentration of 10 μg/ml). In some experiments, brain sections were incubated with a mixture of two EBV pentamers. After extensive washing, sections were fixed in 2% PFA at RT for 15 min and then, after washing in PBS, were incubated overnight with rabbit anti-R-PE polyclonal antibody (1:400 in PBS containing 5% NGS) (GeneTex Inc., Irvine, CA). After washing in PBS, sections were incubated in PBS containing 0.1% H_2_O_2_ for 20 min in the dark to quench the endogenous peroxidase activity and then with biotinylated goat anti-rabbit IgG (1:300 in PBS containing 3% NGS) (Jackson ImmunoResearch Laboratories) for 1 h at RT, followed by avidin-biotin horseradish peroxidase complex using the ABC Vectastain elite kit (Vector Laboratories, Burlingame, CA) for 1 h. The staining reaction was performed with 3,3′-diaminobenzidine (Sigma-Aldrich) as the chromogen. Negative controls included the use of HLA-A or HLA-B mismatched pentamers and omission of anti-R-PE polyclonal antibody. Sections were counterstained with hematoxylin, dehydrated in ethanol, sealed with Canadian balsam, and viewed under an Axiophot microscope (Carl Zeiss, Jena) equipped with a digital camera (AxioCam MRC); images were acquired using AxioVision 4 AC software.

For double immunofluorescence stainings with EBV peptide-coupled HLA class I pentamers and antibodies to CD8, CD20, CD107a, or LMP2A, brain sections were incubated with one or more pentamers and, after fixation with 2% PFA and washing in PBS, with a mixture of rabbit anti-R-PE polyclonal antibody and one of the following MAbs: anti-CD8 mouse MAb (1:50, clone C8/144B; DakoCytomation), anti-CD20 mouse MAb (1:50, clone L26; DakoCytomation), anti-CD107a mouse MAb (1:150; clone H4A3; BD Bioscience, San Jose, CA), and anti-LMP2A rat MAb (1:50) in PBS with 5% NGS. After extensive washing in PBS, sections were incubated with a mixture of biotinylated goat anti-rabbit Ig and Alexa Fluor 488-conjugated goat anti-mouse or donkey anti-rat IgG (Invitrogen) diluted 1:300 in PBS containing 3% NGS or donkey serum and then with TRITC-conjugated streptavidin (Jackson ImmunoResearch Laboratories). After further washings, sections were mounted with antifade mounting medium containing DAPI. Sections were analyzed and images acquired with a digital epifluorescence microscope (Leica Microsystem, Wetzlar, Germany). Negative-control stainings were performed using mismatched pentamers and Ig isotype controls or rabbit preimmune serum.

### Cell counts.

Following careful microscopic examination of brain sections, CD8^+^ cells and pentamer-binding cells were counted in bright field in the whole section area (median value, 3 cm^2^; range, 2.5 to 4.4 cm^2^) with a Plan-Neofluar 20× objective. The percentage of pentamer-binding cells in the total population of brain infiltrating CD8^+^ cells was calculated by dividing the number of pentamer-binding cells counted in a given section by the total number of CD8^+^ cells counted in a serial section that was no more than 5 sections apart.

### Statistical analysis.

For univariate analyses, Mann-Whitney U test (for unpaired data) was used. Multiple independent variables were analyzed by Kruskal-Wallis test with the Dunn-Bonferroni *post hoc* correction for multiple testing. Analyses were performed using IBM SPSS Statistics 25 software.

## References

[1] Compston A, Coles A 2008 Multiple sclerosis. Lancet 372:1502–1517. doi:10.1016/S0140-6736(08)61620-7. 18970977

[2] Olsson T, Barcellos LF, Alfredsson L 2017 Interactions between genetic, lifestyle and environmental risk factors for multiple sclerosis. Nat Rev Neurol 13:25–36. doi:10.1038/nrneurol.2016.187. 27934854

[3] Baranzini SE, Oksenberg JR 2017 The genetics of multiple sclerosis: from 0 to 200 in 50 years. Trends Genet 33:960–970. doi:10.1016/j.tig.2017.09.004. 28987266 PMC5701819

[4] Ascherio A 2013 Environmental factors in multiple sclerosis. Expert Rev Neurother 13:3–9. doi:10.1586/14737175.2013.865866. 24289836

[5] Lucas RM, Hughes AM, Lay ML, Ponsonby AL, Dwyer DE, Taylor BV, Pender MP 2011 Epstein-Barr virus and multiple sclerosis. J Neurol Neurosurg Psychiatry 82:1142–1148. doi:10.1136/jnnp-2011-300174. 21836034

[6] Thorley-Lawson DA 2015 EBV persistence–introducing the virus. Curr Top Microbiol Immunol 390:151–209. doi:10.1007/978-3-319-22822-8_8. 26424647 PMC5125397

[7] Taylor GS, Long HM, Brooks JM, Rickinson AB, Hislop AD 2015 The immunology of Epstein-Barr virus-induced disease. Annu Rev Immunol 33:787–821. doi:10.1146/annurev-immunol-032414-112326. 25706097

[8] Sumaya CV, Myers LW, Ellison GW, Ench Y 1985 Increased prevalence and titer of Epstein-Barr virus antibodies in patients with multiple sclerosis. Ann Neurol 17:371–377. doi:10.1002/ana.410170412. 2988410

[9] Lünemann JD, Huppke P, Roberts S, Brück W, Gärtner J, Münz C 2008 Broadened and elevated humoral immune response to EBNA1 in pediatric multiple sclerosis. Neurology 71:1033–1035. doi:10.1212/01.wnl.0000326576.91097.87. 18809840 PMC2676958

[10] Pakpoor J, Giovannoni G, Ramagopalan SV 2013 Epstein-Barr virus and multiple sclerosis: association or causation? Expert Rev Neurother 13:287–297. doi:10.1586/ern.13.6. 23448218

[11] Handel AE, Williamson AJ, Disanto G, Handunnetthi L, Giovannoni G, Ramagopalan SV 2010 An updated meta-analysis of risk of multiple sclerosis following infectious mononucleosis. PLoS One 5:e12496. doi:10.1371/journal.pone.0012496. 20824132 PMC2931696

[12] Levin LI, Munger KL, O'Reilly EJ, Falk KI, Ascherio A 2010 Primary infection with the Epstein-Barr virus and risk of multiple sclerosis. Ann Neurol 67:824–830. doi:10.1002/ana.21978. 20517945 PMC3089959

[13] Sundström P, Juto P, Wadell G, Hallmans G, Svenningsson A, Nyström L, Dillner J, Forsgren L 2004 An altered immune response to Epstein-Barr virus in multiple sclerosis: a prospective study. Neurology 62:2277–2282. doi:10.1212/01.wnl.0000130496.51156.d7. 15210894

[14] Levin LI, Munger KL, Rubertone MV, Peck CA, Lennette ET, Spiegelman D, Ascherio A 2005 Temporal relationship between elevation of Epstein-Barr virus antibody titers and initial onset of neurological symptoms in multiple sclerosis. JAMA 293:2496–2500. doi:10.1001/jama.293.20.2496. 15914750

[15] DeLorenze GN, Munger KL, Lennette ET, Orentreich N, Vogelman JH, Ascherio A 2006 Epstein-Barr virus and multiple sclerosis: evidence of association from a prospective study with long-term follow-up. Arch Neurol 63:839–844. doi:10.1001/archneur.63.6.noc50328. 16606758

[16] Höllsberg P, Hansen HJ, Haahr S 2003 Altered CD8+ T cell responses to selected Epstein-Barr virus immunodominant epitopes in patients with multiple sclerosis. Clin Exp Immunol 132:137–143. doi:10.1046/j.1365-2249.2003.02114.x. 12653848 PMC1808679

[17] Lünemann JD, Edwards N, Muraro PA, Hayashi S, Cohen JI, Münz C, Martin R 2006 Increased frequency and broadened specificity of latent EBV nuclear antigen-1-specific T cells in multiple sclerosis. Brain 129:1493–1506. doi:10.1093/brain/awl067. 16569670

[18] Jilek S, Schluep M, Meylan P, Vingerhoets F, Guignard L, Monney A, Kleeberg J, Le Goff G, Pantaleo G, Du Pasquier RA 2008 Strong EBV-specific CD8+ T-cell response in patients with early multiple sclerosis. Brain 131:1712–1721. doi:10.1093/brain/awn108. 18550621

[19] Angelini DF, Serafini B, Piras E, Severa M, Coccia EM, Rosicarelli B, Ruggieri S, Gasperini C, Buttari F, Centonze D, Mechelli R, Salvetti M, Borsellino G, Aloisi F, Battistini L 2013 Increased CD8+ T cell response to Epstein-Barr virus lytic antigens in the active phase of multiple sclerosis. PLoS Pathog 9:e1003220. doi:10.1371/journal.ppat.1003220. 23592979 PMC3623710

[20] Latham LB, Lee MJ, Lincoln JA, Ji N, Forsthuber TG, Lindsey JW 2016 Antivirus immune activity in multiple sclerosis correlates with MRI activity. Acta Neurol Scand 133:17–24. doi:10.1111/ane.12417. 25939660

[21] Pender MP, Csurhes PA, Lenarczyk A, Pfluger CM, Burrows SR 2009 Decreased T cell reactivity to Epstein-Barr virus infected lymphoblastoid cell lines in multiple sclerosis. J Neurol Neurosurg Psychiatry 80:498–505. doi:10.1136/jnnp.2008.161018. 19015225 PMC2663364

[22] Pender MP, Csurhes PA, Burrows JM, Burrows SR 2017 Defective T-cell control of Epstein-Barr virus infection in multiple sclerosis. Clin Transl Immunol 6:e126. doi:10.1038/cti.2016.87. PMC5292561 28197337

[23] Jilek S, Schluep M, Harari A, Canales M, Lysandropoulos A, Zekeridou A, Pantaleo G, Du Pasquier RA 2012 HLA-B7-restricted EBV-specific CD8+ T cells are dysregulated in multiple sclerosis. J Immunol 188:4671–4680. doi:10.4049/jimmunol.1103100. 22461701

[24] Montalban X, Hauser SL, Kappos L, Arnold DL, Bar-Or A, Comi G, de Seze J, Giovannoni G, Hartung HP, Hemmer B, Lublin F, Rammohan KW, Selmaj K, Traboulsee A, Sauter A, Masterman D, Fontoura P, Belachew S, Garren H, Mairon N, Chin P, Wolinsky JS, ORATORIO Clinical Investigators 2017 Ocrelizumab versus placebo in primary progressive multiple sclerosis. N Engl J Med 376:209–220. doi:10.1056/NEJMoa1606468. 28002688

[25] Hauser SL, Bar-Or A, Comi G, Giovannoni G, Hartung HP, Hemmer B, Lublin F, Montalban X, Rammohan KW, Selmaj K, Traboulsee A, Wolinsky JS, Arnold DL, Klingelschmitt G, Masterman D, Fontoura P, Belachew S, Chin P, Mairon N, Garren H, Kappos L, OPERA I and OPERA II Clinical Investigators 2017 Ocrelizumab versus interferon beta-1a in relapsing multiple sclerosis. N Engl J Med 376:221–234. doi:10.1056/NEJMoa1601277. 28002679

[26] Sheridan C 2015 Anti-CD20 antibody wows in multiple sclerosis. Nat Biotechnol 33:1215–1216. doi:10.1038/nbt1215-1215. 26649998

[27] Baker D, Pryce G, Amor S, Giovannoni G, Schmierer K 2018 Learning from other autoimmunities to understand targeting of B cells to control multiple sclerosis. Brain 141:2834–2847. doi:10.1093/brain/awy239. 30212896

[28] Lang HL, Jacobsen H, Ikemizu S, Andersson C, Harlos K, Madsen L, Hjorth P, Sondergaard L, Svejgaard A, Wucherpfennig K, Stuart DI, Bell JI, Jones EY, Fugger L 2002 A functional and structural basis for TCR cross-reactivity in multiple sclerosis. Nat Immunol 3:940–943. doi:10.1038/ni835. 12244309

[29] Lünemann JD, Jelcić I, Roberts S, Lutterotti A, Tackenberg B, Martin R, Münz C 2008 EBNA1-specific T cells from patients with multiple sclerosis cross react with myelin antigens and co-produce IFN-gamma and IL-2. J Exp Med 205:1763–1773. doi:10.1084/jem.20072397. 18663124 PMC2525578

[30] van Sechel AC, Bajramovic JJ, van Stipdonk MJ, Persoon-Deen C, Geutskens SB, van Noort JM 1999 EBV-induced expression and HLA-DR-restricted presentation by human B cells of alpha B-crystallin, a candidate autoantigen in multiple sclerosis. J Immunol 162:129–135. 9886378

[31] Pender MP 2003 Infection of autoreactive B lymphocytes with EBV, causing chronic autoimmune diseases. Trends Immunol 24:584–588. doi:10.1016/j.it.2003.09.005. 14596882

[32] Hauser SL, Waubant E, Arnold DL, Vollmer T, Antel J, Fox RJ, Bar-Or A, Panzara M, Sarkar N, Agarwal S, Langer-Gould A, Smith CH, HERMES Trial Group 2008 B-cell depletion with rituximab in relapsing-remitting multiple sclerosis. N Engl J Med 358:676–688. doi:10.1056/NEJMoa0706383. 18272891

[33] Tracy SI, Kakalacheva K, Lünemann JD, Luzuriaga K, Middeldorp J, Thorley-Lawson DA 2012 Persistence of Epstein-Barr virus in self-reactive memory B cells. J Virol 86:12330–12340. doi:10.1128/JVI.01699-12. 22951828 PMC3486485

[34] Panikkar A, Smith C, Hislop A, Tellam N, Dasari V, Hogquist KA, Wykes M, Moss DJ, Rickinson A, Balfour HH Jr, Khanna R 2015 Impaired Epstein-Barr virus-specific neutralizing antibody response during acute infectious mononucleosis is coincident with global B-cell dysfunction. J Virol 89:9137–9141. doi:10.1128/JVI.01293-15. 26109734 PMC4524077

[35] Serafini B, Rosicarelli B, Franciotta D, Magliozzi R, Reynolds R, Cinque P, Andreoni L, Trivedi P, Salvetti M, Faggioni A, Aloisi F 2007 Dysregulated Epstein-Barr virus infection in the multiple sclerosis brain. J Exp Med 204:2899–2912. doi:10.1084/jem.20071030. 17984305 PMC2118531

[36] Serafini B, Severa M, Columba-Cabezas S, Rosicarelli B, Veroni C, Chiappetta G, Magliozzi R, Reynolds R, Coccia EM, Aloisi F 2010 Epstein-Barr virus latent infection and BAFF expression in B-cells in the multiple sclerosis brain: implications for viral persistence and intrathecal B-cell activation. J Neuropathol Exp Neurol 69:677–693. doi:10.1097/NEN.0b013e3181e332ec. 20535037

[37] Serafini B, Muzio L, Rosicarelli B, Aloisi F 2013 Radioactive *in situ* hybridization for EBER supports presence of Epstein-Barr virus in the multiple sclerosis brain. Brain 136:e233. doi:10.1093/brain/aws315. 23355688

[38] Magliozzi R, Serafini B, Rosicarelli B, Chiappetta G, Veroni C, Reynolds R, Aloisi F 2013 B-cell enrichment and Epstein-Barr virus infection in inflammatory cortical lesions in secondary progressive multiple sclerosis. J Neuropathol Exp Neurol 72:29–41. doi:10.1097/NEN.0b013e31827bfc62. 23242282

[39] Veroni C, Serafini B, Rosicarelli B, Fagnani C, Aloisi F 2018 Transcriptional profile and Epstein-Barr virus infection status of laser-cut immune infiltrates from the brain of patients with progressive multiple sclerosis. J Neuroinflammation 15:18. doi:10.1186/s12974-017-1049-5. 29338732 PMC5771146

[40] Tzartos JS, Khan G, Vossenkamper A, Cruz-Sadaba M, Lonardi S, Sefia E, Meager A, Elia A, Middeldorp JM, Clemens M, Farrell PJ, Giovannoni G, Meier UC 2012 Association of innate immune activation with latent Epstein-Barr virus in active MS lesions. Neurology 78:15–23. doi:10.1212/WNL.0b013e31823ed057. 22156987

[41] Hassani A, Corboy JR, Al-Salam S, Khan G 2018 Epstein-Barr virus is present in the brain of most cases of multiple sclerosis and may engage more than just B cells. PLoS One 13:e0192109. doi:10.1371/journal.pone.0192109. 29394264 PMC5796799

[42] Moreno MA, Or-Geva N, Aftab BT, Khanna R, Croze E, Steinman L, Han MH 2018 Molecular signature of Epstein-Barr virus infection in MS brain lesions. Neurol Neuroimmunol Neuroinflamm 5:e466. doi:10.1212/NXI.0000000000000466. 29892607 PMC5994704

[43] Willis SN, Stadelmann C, Rodig SJ, Caron T, Gattenloehner S, Mallozzi SS, Roughan JE, Almendinger SE, Blewett MM, Brück W, Hafler DA, O'Connor KC 2009 Epstein-Barr virus infection is not a characteristic feature of multiple sclerosis brain. Brain 132:3318–3328. doi:10.1093/brain/awp200. 19638446 PMC2792367

[44] Peferoen LA, Lamers F, Lodder LN, Gerritsen WH, Huitinga I, Melief J, Giovannoni G, Meier U, Hintzen RQ, Verjans GM, van Nierop GP, Vos W, Peferoen-Baert RM, Middeldorp JM, van der Valk P, Amor S 2010 Epstein Barr virus is not a characteristic feature in the central nervous system in established multiple sclerosis. Brain 133:e137. doi:10.1093/brain/awp296. 19917644

[45] Sargsyan SA, Shearer AJ, Ritchie AM, Burgoon MP, Anderson S, Hemmer B, Stadelmann C, Gattenlöhner S, Owens GP, Gilden D, Bennett JL 2010 Absence of Epstein-Barr virus in the brain and CSF of patients with multiple sclerosis. Neurology 74:1127–1135. doi:10.1212/WNL.0b013e3181d865a1. 20220124 PMC2865779

[46] Torkildsen Ø, Stansberg C, Angelskår SM, Kooi EJ, Geurts JJ, van der Valk P, Myhr KM, Steen VM, Bø L 2010 Upregulation of immunoglobulin-related genes in cortical sections from multiple sclerosis patients. Brain Pathol 20:720–729. doi:10.1111/j.1750-3639.2009.00343.x. 19919606 PMC8094770

[47] Aloisi F, Serafini B, Magliozzi R, Howell OW, Reynolds R 2010 Detection of Epstein-Barr virus and B-cell follicles in the multiple sclerosis brain: what you find depends on how and where you look. Brain 133:e157. doi:10.1093/brain/awq223. 20739348

[48] Lassmann H, Niedobitek G, Aloisi F, Middeldorp JM, NeuroproMiSe EBV Working Group 2011 Epstein-Barr virus in the multiple sclerosis brain: a controversial issue–report on a focused workshop held in the Centre for Brain Research of the Medical University of Vienna, Austria. Brain 134:2772–2786. doi:10.1093/brain/awr197. 21846731 PMC3170536

[49] Hislop AD, Taylor GS 2015 T-cell responses to EBV. Curr Top Microbiol Immunol 391:325–353. doi:10.1007/978-3-319-22834-1_11. 26428380

[50] Hayashi T, Morimoto C, Burks JS, Kerr C, Hauser SL 1988 Dual-label immunocytochemistry of the active multiple sclerosis lesion: major histocompatibility complex and activation antigens. Ann Neurol 24:523–531. doi:10.1002/ana.410240408. 3266456

[51] Denic A, Wootla B, Rodriguez M 2013 CD8+ T cells in multiple sclerosis. Expert Opin Ther Targets 17:1053–1066. doi:10.1517/14728222.2013.815726. 23829711 PMC3928018

[52] Babbe H, Roers A, Waisman A, Lassmann H, Goebels N, Hohlfeld R, Friese M, Schröder R, Deckert M, Schmidt S, Ravid R, Rajewsky K 2000 Clonal expansions of CD8(+) T cells dominate the T cell infiltrate in active multiple sclerosis lesions as shown by micromanipulation and single cell polymerase chain reaction. J Exp Med 192:393–404. doi:10.1084/jem.192.3.393. 10934227 PMC2193223

[53] van Nierop GP, van Luijn MM, Michels SS, Melief MJ, Janssen M, Langerak AW, Ouwendijk WJD, Hintzen RQ, Verjans G 2017 Phenotypic and functional characterization of T cells in white matter lesions of multiple sclerosis patients. Acta Neuropathol 134:383–401. doi:10.1007/s00401-017-1744-4. 28624961 PMC5563341

[54] Jaquiéry E, Jilek S, Schluep M, Meylan P, Lysandropoulos A, Pantaleo G, Du Pasquier RA 2010 Intrathecal immune responses to EBV in early MS. Eur J Immunol 40:878–887. doi:10.1002/eji.200939761. 20017197

[55] Lossius A, Johansen JN, Vartdal F, Robins H, Jūratė Šaltytė B, Holmøy T, Olweus J 2014 High-throughput sequencing of TCR repertoires in multiple sclerosis reveals intrathecal enrichment of EBV-reactive CD8+ T cells. Eur J Immunol 44:3439–3452. doi:10.1002/eji.201444662. 25103993

[56] van Nierop GP, Janssen M, Mitterreiter JG, van de Vijver DA, de Swart RL, Haagmans BL, Verjans GM, Hintzen RQ 2016 Intrathecal CD8 T-cells of multiple sclerosis patients recognize lytic Epstein-Barr virus proteins. Mult Scler 22:279–291. doi:10.1177/1352458515588581. 26041797

[57] van Nierop GP, Janssen M, Mitterreiter JG, van de Vijver DA, de Swart RL, Haagmans BL, Verjans GM, Hintzen RQ 2016 Intrathecal CD4(+) and CD8(+) T-cell responses to endogenously synthesized candidate disease-associated human autoantigens in multiple sclerosis patients. Eur J Immunol 46:347–353. doi:10.1002/eji.201545921. 26507805

[58] Serafini B, Scorsi E, Rosicarelli B, Rigau V, Thouvenot E, Aloisi F 2017 Massive intracerebral Epstein-Barr virus reactivation in lethal multiple sclerosis relapse after natalizumab withdrawal. J Neuroimmunol 307:14–17. doi:10.1016/j.jneuroim.2017.03.013. 28495131

[59] Serafini B, Zandee S, Rosicarelli B, Scorsi E, Veroni C, Larochelle C, D'Alfonso S, Prat A, Aloisi F 2018 Epstein-Barr virus-associated immune reconstitution inflammatory syndrome as possible cause of fulminant multiple sclerosis relapse after natalizumab interruption. J Neuroimmunol 319:9–12. doi:10.1016/j.jneuroim.2018.03.011. 29685294

[60] Coppieters KT, Dotta F, Amirian N, Campbell PD, Kay TW, Atkinson MA, Roep BO, von Herrath MG 2012 Demonstration of islet-autoreactive CD8 T cells in insulitic lesions from recent onset and long-term type 1 diabetes patients. J Exp Med 209:51–60. doi:10.1084/jem.20111187. 22213807 PMC3260877

[61] van Velzen M, Jing L, Osterhaus AD, Sette A, Koelle DM, Verjans GM 2013 Local CD4 and CD8 T-cell reactivity to HSV-1 antigens documents broad viral protein expression and immune competence in latently infected human trigeminal ganglia. PLoS Pathog 9:e1003547. doi:10.1371/journal.ppat.1003547. 23966859 PMC3744444

[62] Hislop AD, Taylor GS, Sauce D, Rickinson AB 2007 Cellular responses to viral infection in humans: lessons from Epstein-Barr virus. Annu Rev Immunol 25:587–617. doi:10.1146/annurev.immunol.25.022106.141553. 17378764

[63] Pudney VA, Leese AM, Rickinson AB, Hislop AD 2005 CD8+ immunodominance among Epstein-Barr virus lytic cycle antigens directly reflects the efficiency of antigen presentation in lytically infected cells. J Exp Med 201:349–360. doi:10.1084/jem.20041542. 15684323 PMC2213038

[64] Nozuma S, Jacobson S 2019 Neuroimmunology of human T-lymphotropic virus type 1-associated myelopathy/tropical spastic paraparesis. Front Microbiol 10:885. doi:10.3389/fmicb.2019.00885. 31105674 PMC6492533

[65] Serafini B, Rosicarelli B, Aloisi F, Stigliano E 2014 EBV in the central nervous system and cervical lymph node of a patient with primary progressive multiple sclerosis. J Neuropathol Exp Neurol 73:729–731. doi:10.1097/NEN.0000000000000082. 24918642

[66] Petereit HF, Rubbert-Roth A 2009 Rituximab levels in cerebrospinal fluid of patients with neurological autoimmune disorders. Mult Scler 15:189–192. doi:10.1177/1352458508098268. 18971221

[67] Cross AH, Stark JL, Lauber J, Ramsbottom MJ, Lyons JA 2006 Rituximab reduces B cells and T cells in cerebrospinal fluid of multiple sclerosis patients. J Neuroimmunol 180:63–70. doi:10.1016/j.jneuroim.2006.06.029. 16904756 PMC1769354

[68] Piccio L, Naismith RT, Trinkaus K, Klein RS, Parks BJ, Lyons JA, Cross AH 2010 Changes in B- and T-lymphocyte and chemokine levels with rituximab treatment in multiple sclerosis. Arch Neurol 67:707–714. doi:10.1001/archneurol.2010.99. 20558389 PMC2918395

[69] Albanese M, Tagawa T, Buschle A, Hammerschmidt W 2017 MicroRNAs of Epstein-Barr virus control innate and adaptive antiviral immunity. J Virol 91:e01667-16. doi:10.1128/JVI.01667-16. 28592533 PMC5533892

[70] Ressing ME, van Gent M, Gram AM, Hooykaas MJ, Piersma SJ, Wiertz EJ 2015 Immune evasion by Epstein-Barr virus. Curr Top Microbiol Immunol 391:355–381. doi:10.1007/978-3-319-22834-1_12. 26428381

[71] Goodman A, Patel SP, Kurzrock R 2017 PD-1-PD-L1 immune-checkpoint blockade in B-cell lymphomas. Nat Rev Clin Oncol 14:203–220. doi:10.1038/nrclinonc.2016.168. 27805626

[72] Anastasiadou E, Stroopinsky D, Alimperti S, Jiao AL, Pyzer AR, Cippitelli C, Pepe G, Severa M, Rosenblatt J, Etna MP, Rieger S, Kempkes B, Coccia EM, Sui SJH, Chen CS, Uccini S, Avigan D, Faggioni A, Trivedi P, Slack FJ 2019 Epstein-Barr virus-encoded EBNA2 alters immune checkpoint PD-L1 expression by downregulating miR-34a in B-cell lymphomas. Leukemia 33:132–147. doi:10.1038/s41375-018-0178-x. 29946193 PMC6327052

[73] Trivedi P, Slack FJ, Anastasiadou E 2018 Epstein-Barr virus: from kisses to cancer, an ingenious immune evader. Oncotarget 9:36411–36412. doi:10.18632/oncotarget.26381. 30559926 PMC6284857

[74] Cencioni MT, Magliozzi R, Nicholas R, Ali R, Malik O, Reynolds R, Borsellino G, Battistini L, Muraro PA 2017 Programmed death 1 is highly expressed on CD8+ CD57+ T cells in patients with stable multiple sclerosis and inhibits their cytotoxic response to Epstein-Barr virus. Immunology 152:660–676. doi:10.1111/imm.12808. 28767147 PMC5680058

[75] Lycke J 2017 Trials of antivirals in the treatment of multiple sclerosis. Acta Neurol Scand 136:45–48. doi:10.1111/ane.12839. 29068492

[76] Drosu NC, Edelman ER, Housman DE 2018 Could antiretrovirals be treating EBV in MS? A case report. Mult Scler Relat Disord 22:19–21. doi:10.1016/j.msard.2018.02.029. 29510325 PMC6100748

[77] Lin JC, Zhang ZX, Smith MC, Biron K, Pagano JS 1988 Anti-human immunodeficiency virus agent 3’-azido’-3’-deoxythymidine inhibits replication of Epstein-Barr virus. Antimicrob Agents Chemother 32:265–267. doi:10.1128/aac.32.2.265. 2834997 PMC172149

[78] Pender MP, Csurhes PA, Smith C, Douglas NL, Neller MA, Matthews KK, Beagley L, Rehan S, Crooks P, Hopkins TJ, Blum S, Green KA, Ioannides ZA, Swayne A, Aftab BT, Hooper KD, Burrows SR, Thompson KM, Coulthard A, Khanna R 2018 Epstein-Barr virus-specific T cell therapy for progressive multiple sclerosis. JCI Insight 3:124714. doi:10.1172/jci.insight.124714. 30429369 PMC6302936

[79] Sugden B 2014 Epstein-Barr virus: the path from association to causality for a ubiquitous human pathogen. PLoS Biol 12:e1001939. doi:10.1371/journal.pbio.1001939. 25180782 PMC4151957

[80] Cohen JI 2018 Vaccine development for Epstein-Barr virus. Adv Exp Med Biol 1045:477–493. doi:10.1007/978-981-10-7230-7_22. PMC6328312 29896681

[81] International Multiple Sclerosis Genetics Consortium, Wellcome Trust Case Control Consortium 2, Sawcer S, Hellenthal G, Pirinen M, Spencer CCA, Patsopoulos NA, Moutsianas L, Dilthey A, Su Z, Freeman C, Hunt SE, Edkins S, Gray E, Booth DR, Potter SC, Goris A, Band G, Oturai AB, Strange A, Saarela J, Bellenguez C, Fontaine B, Gillman M, Hemmer B, Gwilliam R, Zipp F, Jayakumar A, Martin R, Leslie S, Hawkins S, Giannoulatou E, D'alfonso S, Blackburn H, Martinelli Boneschi F, Liddle J, Harbo HF, Perez ML, Spurkland A, Waller MJ, Mycko MP, Ricketts M, Comabella M, Hammond N, Kockum I, McCann OT, Ban M, Whittaker P, Kemppinen A, Weston P, Hawkins C, Widaa S, Zajicek J, Dronov S, Robertson N, Bumpstead SJ, Barcellos LF, Ravindrarajah R, Abraham R, Alfredsson L, Ardlie K, Aubin C, Baker A, Baker K, Baranzini SE, Bergamaschi L, Bergamaschi R, Bernstein A, Berthele A, Boggild M, Bradfield JP, Brassat D, Broadley SA, Buck D, Butzkueven H, Capra R, Carroll WM, Cavalla P, Celius EG, Cepok S, Chiavacci R, Clerget-Darpoux F, Clysters K, Comi G, Cossburn M, Cournu-Rebeix I, Cox MB, Cozen W, Cree BAC, Cross AH, Cusi D, Daly MJ, Davis E, de Bakker PIW, Debouverie M, D'hooghe MB, Dixon K, Dobosi R, Dubois B, Ellinghaus D, Elovaara I, Esposito F, Fontenille C, Foote S, Franke A, Galimberti D, Ghezzi A, Glessner J, Gomez R, Gout O, Graham C, Grant SFA, Guerini FR, Hakonarson H, Hall P, Hamsten A, Hartung H-P, Heard RN, Heath S, Hobart J, Hoshi M, Infante-Duarte C, Ingram G, Ingram W, Islam T, Jagodic M, Kabesch M, Kermode AG, Kilpatrick TJ, Kim C, Klopp N, Koivisto K, Larsson M, Lathrop M, Lechner-Scott JS, Leone MA, Leppä V, Liljedahl U, Bomfim IL, Lincoln RR, Link J, Liu J, Lorentzen AR, Lupoli S, Macciardi F, Mack T, Marriott M, Martinelli V, Mason D, McCauley JL, Mentch F, Mero I-L, Mihalova T, Montalban X, Mottershead J, Myhr K-M, Naldi P, Ollier W, Page A, Palotie A, Pelletier J, Piccio L, Pickersgill T, Piehl F, Pobywajlo S, Quach HL, Ramsay PP, Reunanen M, Reynolds R, Rioux JD, Rodegher M, Roesner S, Rubio JP, Rückert I-M, Salvetti M, Salvi E, Santaniello A, Schaefer CA, Schreiber S, Schulze C, Scott RJ, Sellebjerg F, Selmaj KW, Sexton D, Shen L, Simms-Acuna B, Skidmore S, Sleiman PMA, Smestad C, Sørensen PS, Søndergaard HB, Stankovich J, Strange RC, Sulonen A-M, Sundqvist E, Syvänen A-C, Taddeo F, Taylor B, Blackwell JM, Tienari P, Bramon E, Tourbah A, Brown MA, Tronczynska E, Casas JP, Tubridy N, Corvin A, Vickery J, Jankowski J, Villoslada P, Markus HS, Wang K, Mathew CG, Wason J, Palmer CNA, Wichmann H-E, Plomin R, Willoughby E, Rautanen A, Winkelmann J, Wittig M, Trembath RC, Yaouanq J, Viswanathan AC, Zhang H, Wood NW, Zuvich R, Deloukas P, Langford C, Duncanson A, Oksenberg JR, Pericak-Vance MA, Haines JL, Olsson T, Hillert J, Ivinson AJ, De Jager PL, Peltonen L, Stewart GJ, Hafler DA, Hauser SL, McVean G, Donnelly P, Compston A 2011 Genetic risk and a primary role for cell-mediated immune mechanisms in multiple sclerosis. Nature 476:214–219. doi:10.1038/nature10251. 21833088 PMC3182531

